# Recent developments in the intervention of specific phobia among adults: a rapid review

**DOI:** 10.12688/f1000research.20082.1

**Published:** 2020-03-19

**Authors:** Christabel E.W. Thng, Nikki S.J. Lim-Ashworth, Brian Z.Q. Poh, Choon Guan Lim

**Affiliations:** 1 Department of Developmental Psychiatry, Institute of Mental Health, 10 Buangkok View, 539747, Singapore

**Keywords:** Specific phobia, intervention, virtual reality, psychological treatment, phobia

## Abstract

Specific phobia is highly prevalent worldwide. Although the body of intervention studies is expanding, there is a lack of reviews that summarise recent progress and discuss the challenges and direction of research in this area. Hence, this rapid review seeks to systematically evaluate the available evidence in the last five years in the treatment of specific phobias in adults. Studies published between January 2014 to December 2019 were identified through searches on the electronic databases of Medline and PsychINFO. In total, 33 studies were included. Evidence indicates that psychotherapy, and in particular cognitive behaviour therapy, when implemented independently or as an adjunctive, is a superior intervention with large effect sizes. Technology-assisted therapies seem to have a beneficial effect on alleviating fears and are described to be more tolerable than *in vivo* exposure therapy. Pharmacological agents are investigated solely as adjuncts to exposure therapy, but the effects are inconsistent; propranolol and glucocorticoid may be promising. A handful of cognitive-based therapies designed to alter fear arousal and activation pathways of phobias have presented preliminary, positive outcomes. Challenges remain with the inherent heterogeneity of specific phobia as a disorder and the accompanying variability in outcome measures and intervention approaches to warrant a clear conclusion on efficacy.

## Recent developments in the intervention of specific phobia among adults: a rapid review

Specific phobia, which has a lifetime prevalence of 7.4%, is one of the most common disorders ^1^. It is defined in the *Diagnostic and Statistical Manual of Mental Disorders*, 5th edition (DSM-5) as a marked fear or anxiety about a specific object or situation (for example, flying, heights, animals, receiving an injection, or seeing blood) ^2^. The four subtypes recognised in the DSM-5 are animal (for example, spiders and insects), natural environment (for example, heights and storms), blood-injection-injury (for example, needles and invasive medical procedures) and situational (for example, airplanes, elevators and closed spaces), and there is an ‘other’ category for phobias that do not fit into the aforementioned subtypes. The phobic object or situation almost always provokes immediate fear or anxiety and is actively avoided or endured with intense fear or anxiety. This fear or anxiety is out of proportion to the actual danger posed by the specific object or situation and to the sociocultural context. These symptoms typically last for 6 months or more and can cause clinically significant distress or impairment in social, occupational or other important areas of functioning ^2^.

To date, the treatment of specific phobia revolves around the following: pharmacological therapy, behavioural therapy and cognitive therapy. In particular, *in vivo* exposure appeared to be the most efficacious intervention for a wide variety of phobias, and a few studies obtained a response rate of 80 to 90%, based on a review by Choy *et al*. ^3^. Other forms of exposure approaches for specific phobia include imaginal exposure and systematic desensitisation ^4– 6^ and eye movement desensitisation and reprocessing (EMDR) ^7^. These exposure interventions have demonstrated varying levels of treatment success with different types of specific phobias ^3^. More recently, the use of technology and virtual reality (VR) as part of therapy has become more commonplace ^8, 9^.

In 2007, Choy *et al*. completed a comprehensive review of the treatment studies in specific phobias ^3^. The review evaluated acute and long-term efficacy studies of *in vivo* exposure, VR, cognitive therapy and other treatments from 1960 to 2005. Since then, many more studies have been carried out in this area, offering us new insights into the treatment of specific phobias.

Thus, the present study aims to systematically examine the research that has been carried out on the treatment of specific phobias in the past five years and to provide an update on the latest literature by using a rapid review methodology. We have categorised the studies into those that focus on (1) psychological treatments, (2) involving the use of technology, (3) pharmacological treatments and (4) others (for studies that do not fall under the first three categories).

## Methods

**Figure 1.**
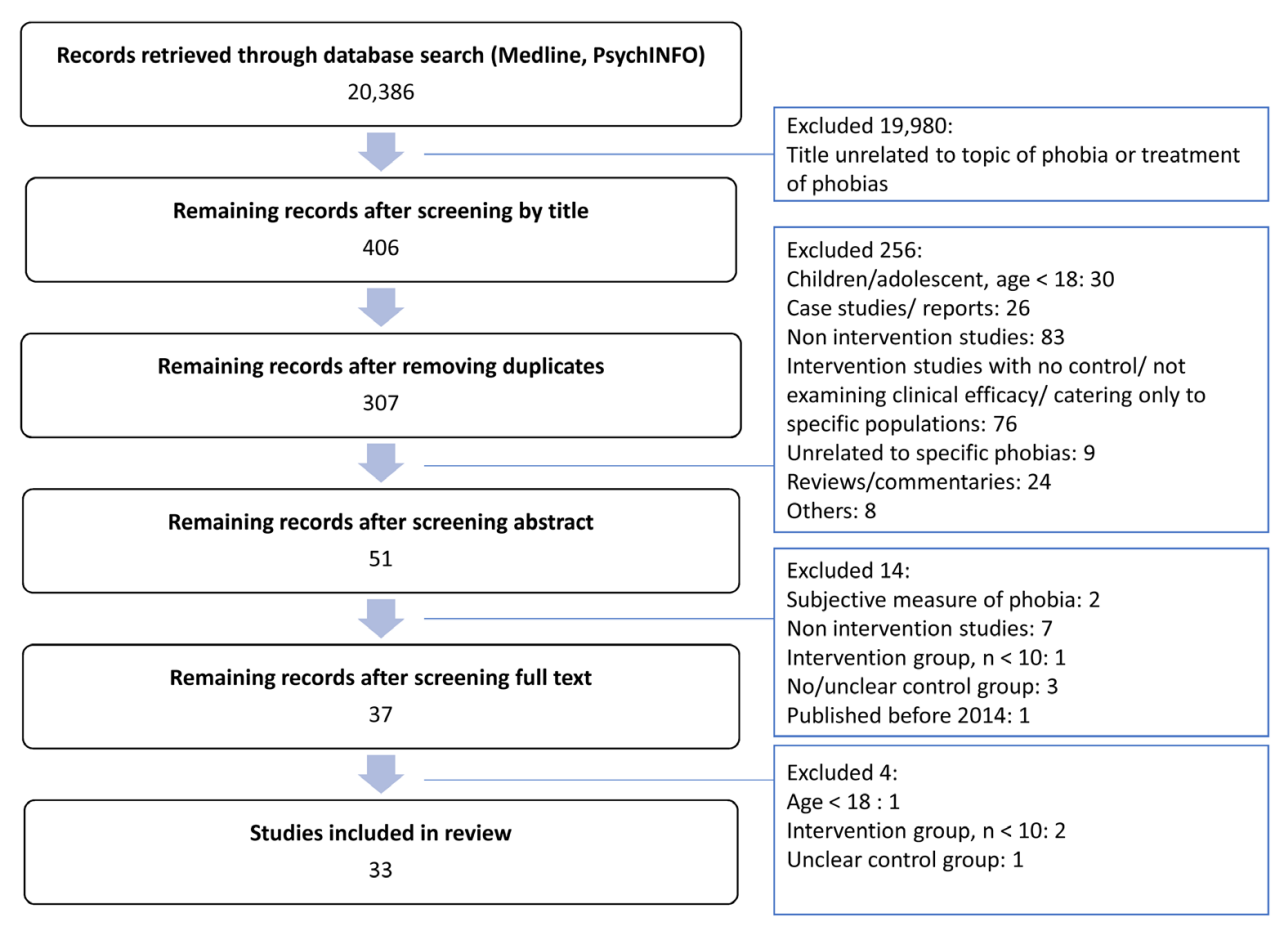
Information about study screening, selection and exclusion.

A computer search of PsychINFO and Medline from January 2014 to December 2019 was conducted by using the following search terms: “phob*”, “phobia”, “fear”, “phobic”, “phobic neurosis” and “phobic neuroses”. These were combined with “treatment”, “therapy”, “psychological therapy”, “psychotherapy”, “supportive therapy”, “drug”, “pharmacotherapy” and “medication”. The titles and abstracts of this comprehensive search were reviewed, and articles were selected on the basis of the inclusion and exclusion criteria listed below. Two reviewers independently reviewed the full text of the selected articles to decide on the final list of studies to be included. Any discrepancies in the selection of articles were discussed and resolved between the two reviewers. The overview of the search process is presented in Figure 1.

Studies were included if they met the following criteria:

1. Published in the English language between January 2014 and December 2019

2. Adults 18 years old or older (If age range was not reported, we included articles that reported a mean age of 18 years or older.)

3. Sample size of at least 10 in the intervention group

4. Studies looking at specific phobias or fears

5. Study designs that included a control and compared at least two treatments in parallel.

We excluded the following types of studies:

1. Studies that controlled only for variations of a treatment component (for example, importance of therapist involvement and importance of relaxation) and did not have a separate control group or active treatment

2. Studies with crossover designs, because treatment effects can be confounded by carry-over and learning effects depending on the order in which treatment is administered

3. Studies with analogue samples, as these would not be representative of the general population (that is, studies that had used samples that might not be representative of the clinical population; for example, individuals who are not evaluated using a clinical tool or a structured assessment process, and the severity of their functioning or the level of motivation may differ from that of the actual clinical population).

## Results

### Overall study characteristics

In all, 33 studies fulfilled the selection benchmarks and were included in this review. Table 1 presents a detailed summary of the included studies. The mean age of the participants from the studies included in the rapid review range from 19.12 ^10^ to 65.72 ^11^. Across the studies, sample sizes were generally smaller for studies with clinical sample and bigger for community studies, varying from 22 ^12^ to 371 ^13^, and the median was 60. The majority of the studies had community-based samples. Attrition rates range from 0 to 46% and the median was 3.3%. Loss to follow-up of up to 5% is of minimum concern, whereas loss of more than 20% may be a source of bias in randomised controlled trials ^14^. Five studies included in this review have attrition rates of more than 20% ^10, 13, 15– 17^, but they are of survey research design, which comes with an expected high attrition rate ^18^. There are far more female than male participants in many of the studies, especially for those that focused on fear of spiders (13 studies), needles/injections (two studies) and heights/flying (eight studies). These fears tend to be more pervasive in females than males ^19^. Three studies that examined the fear of childbirth naturally restricted the samples to female participants. Last but not least, the follow-up periods range from immediate follow-up to 1 year, and the median is 1 month.

**Table 1.** Characteristics of studies examining the efficacy of an intervention for specific phobias.

	Source	Type of treatment	Specific phobia type and sample origin	Treatment condition and sample size	Age range (M)	Format of delivery	Primary outcome measure	Outcomes
1	Cai *et al*., 2018 ^11^	Psychotherapy	Kinesiophobia (community)	1: CBT (psychoeducation and relaxation) (n = 50) 2: Standard care control (n = 50)	57–74 (65.72)	4 sessions - 30 min	- TSK	Post: 1 > 2 6 months: 1 > 2
2	Cougle *et al*., 2016 ^10^	Psychotherapy	Spiders (community)	1: Exposure to contamination (n = 17) 2: Wait-list control (n = 17)	Not stated (19.12)	3 sessions - 30 min - over 2 weeks	- BAT - DPSS-12 - FSQ	1 week: 1 > 2 Moderated by high levels of general disgust propensity at pre- treatment
3	Klabbers *et al*., 2019 ^15^	Psychotherapy	Fear of childbirth (community)	1: Haptotherapy (n = 51) 2: Psycho-education via the Internet (n = 39) 3: Care as usual (n = 44)	Not stated (32)	8 sessions (haptotherapy) - 60 min 8 modules (psychoeducation) - over 8 weeks	- TES - W-DEQ - 4DSQ	6 months: 1 > 2 and 3
4	Kowalsky *et al*., 2018 ^23^	Psychotherapy	Fear of needles (community)	1: AMT-RI; applied muscle tension (AMT) during trial one and respiratory intervention (RI) during trial two (n = 31) 2: RI-AMT; RI during trial one and AMT during trial two (n = 33) 3: No treatment control (n = 31)	19–55 (23.4)	1 session	- MFS – Needle and Blood subscales	1 > 2 = 3
5	Matthews *et al*., 2015 ^24^	Psychotherapy	Spiders (community)	1: Real exposure (online) (n = 14) 2: Hyper-real exposure (online) (n = 11) 3: Control exposure (n = 9)	Not stated (26.5)	8 sessions - over 1 week	- FSQ - BAT - CIDI- specific phobia module	1 month: 1 = 2 > 3
6	Riddle- Walker *et al*., 2016 ^21^	Psychotherapy	Emetophobia (clinical)	1: CBT with exposure (n = 12) 2: Wait-list control (n = 12)	Not stated (33.5)	12 sessions - 60 min - over 18 weeks	- EmetQ - HAI - ASI - PHQ-9 - SDS	Post: 1 > 2 2 months: 1 > 2
7	Rondung *et al*., 2018 ^16^	Psychotherapy	Fear of childbirth (community)	1: CBT (internet-based) (n = 127) 2: Standard care, counselling by midwives (n = 131)	17–42 (29.6)	7 modules - over 8 weeks	- FOBS	Post: 1 = 2 1 year: 1 > 2 Findings not easily interpreted due to limitations
8	Rouhe *et al*., 2015 ^13^	Psychotherapy	Fear of childbirth (community)	1: Group psychoeducation with relaxation (n = 131) 2: Control group (n = 240)	Not stated	6 sessions - 120 min - group	- MAMA - W-DEQ - EPDS - TES	3 months: 1 > 2
9	Siegel and Gallagher, 2015 ^25^	Psychotherapy	Spiders (community)	1: Very brief exposure (VBE), 24-hour BAT (n = 22) 2: Very brief exposure (VBE), immediate BAT (n = 21) 3: Control exposure, 24-hour BAT (n = 21) 4: Control exposure, immediate BAT (n = 22)	Not stated (19.4)	2 sessions - over 1 week	- FSQ - BAT	1 day: 1 = 2 > 3 = 4
10	Tellez *et al*., 2015 ^17^	Psychotherapy	Dental anxiety (community)	1: CBT with psychoeducation, exposure (computer- based) (n = 74) 2: Wait-list control group (n = 77)	18–70 (44.7)	1 session - 60 min	- MDAS	1 month: 1 > 2
11	Triscari *et al*., 2015 ^22^	Psychotherapy	Flying (clinical)	1: CBT with systematic desensitization (CBT-SD) (n = 22) 2: CBT with eye movement desensitization and reprocessing therapy (CBT-EMDR) (n = 22) 3: CBT with virtual reality exposure therapy (CBT-VRET) (n = 21)	24–70 (43.52)	10 sessions - 120 min	- FAM - FAS	Post: 1 = 2 = 3 1 year: 1 = 2 = 3
12	Botella *et al*., 2016 ^27^	Technology- assisted therapy	Spiders/ cockroaches (community)	1: AR (n = 32) 2: *In vivo* exposure (n = 31)	20–70 (31.73)	1 session - 180 min - individual	- BAT	Post: 1 < 2 12 weeks: 1 = 2 24 weeks: 1 = 2
13	Freeman *et al*., 2018 ^28^	Technology- assisted therapy	Heights (community)	1: VRET (n = 49) 2: TAU (n = 51)	30–58 (45.51)	6 sessions - 30 min - individual	- HIQ	Post: 1 > 2 4 weeks: 1 > 2
14	Gujjar *et al*., 2019 ^29^	Technology- assisted therapy	Dental anxiety (clinical)	1: VRET (n = 15) 2: Psychoeducation with information pamphlet (n = 15)	18–50 (24.15)	1 session - 40 min - individual	- VAS-A - MDAS - DFS	Post: 1 > 2 1 week: 1 > 2 12 weeks: 1 > 2 24 weeks: 1 > 2
15	Lima *et al*., 2018 ^30^	Technology- assisted therapy	Storms (community)	1: VRET (n = 18) 2: Progressive muscle relaxation + psychoeducation (n = 18)	Not stated (27.56)	1 session - 60 min - individual	- SFQ - BAT	Post: 1 > 2 4 weeks: 1 = 2
16	Miloff *et al*., 2019 ^31^	Technology- assisted therapy	Spiders (community)	1: VRET ( *VIMSE*) (n = 50) 2: *In vivo* exposure (n = 50)	>18 (34.05)	1 session - 180 min - individual	- BAT	Post: 1 < 2 12 weeks: 1 = 2 1 year: 1 = 2
17	Minns *et al*., 2019 ^32^	Technology- assisted therapy	Spiders (community)	1: VRET (n = 38) 2: Wait list (n = 39)	18–65 (19.27)	1 session - 30 min - individual	- FSQ	Post: 1 > 2
18	Yilmaz Yelvar *et al*., 2017 ^33^	Technology- assisted therapy	Kinesiophobia (community)	1: VRET physiotherapy (n = 23) 2: Traditional physiotherapy (n = 23)	Not stated (49.54)	10 sessions - individual	- TSK	Post: 1 > 2
19	Acheson *et al*., 2015 ^34^	Pharmacotherapy	Spiders (community)	1: Exposure + 24 IU oxytocin (n = 10) 2: Exposure + placebo (n = 13)	Not stated (1. 26.8, 2. 33.3)	1 session	- SPQ - FSQ - BAT	1 month: 2 > 1
20	Meyerbröker *et al*., 2018 ^35^	Pharmacotherapy	Heights/flying (clinical)	1: VRET + 15 mg yohimbine (n = 18) 2: VRET + placebo (n = 18) 3: VRET + 40 mg propranolol (n = 18)	19–65 (36.6)	3 sessions - over 2 weeks	- WAQ - AQ - ATHQ - FAM - FAS	3 months: 1 = 2 = 3
21	Raeder *et al*., 2019 ^36^	Pharmacotherapy	Spiders (community)	1: Exposure + 20 mg hydrocortisone (n = 20) 2: Exposure + placebo (n = 23)	18–65 (1. 22.7, 2. 22.6)	1 session	- SPQ - FSQ - SBQ - BAT	7 months: 2 > 1
22	Soeter and Kindt, 2015 ^37^	Pharmacotherapy	Spiders (community)	1: Exposure + 40 mg propranolol (n = 15) 2: Exposure + placebo (n = 15) 3: 40 mg Propranolol (n = 15)	18–32 (21.6)	1 session	- SPQ - BAT	1 year: 1 > 2 = 3
23	Soravia *et al*., 2014 ^12^	Pharmacotherapy	Spiders (community)	1: VRET + 20 mg hydrocortisone (n = 11) 2: VRET + Placebo (n = 11)	20–60 (33.1)	2 sessions - group - over 2 weeks	- FSQ	1 month: 1 > 2
24	Telch *et al*., 2014 ^38^	Pharmacotherapy	Enclosed space (community)	1: Extinction training + 260 mg methylene blue 2: Extinction training + placebo	18–36 (19.3)	1 session	- BAT	1 month: 2 > 1
25	Dreyer-Oren *et al*., 2019 ^39^	Others	Heights (community)	1: CBM (n = 27) 2: CBM + exposure (n = 26) 3: Exposure (n = 26) 4: Control (sham CBM) (n = 28)	18–67 (23.58)	2 sessions - 90 min - individual	- AQ-anxiety - HIQ	Post: 3 > 1 > 2 > 4
26	Maples- Keller *et al*., 2017 ^40^	Others	Flying (community)	1: Reactivation cue prior to VRE (n = 45) 2: Neutral cue prior to VRE (n = 44)	21–67 (42.11)	4 sessions - individual	- FFI - STAI	Post: 1 = 2 3 months: 1 = 2 1 year: 1 = 2
27	Meuret *et al*., 2017 ^41^	Others	Blood-injection- injury	1: Symptom-associated tension training (SAT) (n = 20) 2: Hypoventilation Respiratory training - HRT (n = 20) 3: Relaxation skills training (RST) (n = 20)	18–49 (27.9)	1 session - 12 min - individual	- MFS	Post: 1 and 2 > 3
28	Notzon *et al*., 2015 ^42^	Others	Spiders (community)	1: iTBS in spider phobics (n = 21), in healthy controls (n = 19) 2: Sham in spider phobics (n = 20), in healthy controls (n = 23)	18–65	1 session - 3 min - individual	- FSQ - SUDS - HR - SCL	Post: 1 = 2
29	Seinfeld *et al*., 2016 ^43^	Others	Heights (community)	1: VRE with background music (n = 20) 2: VRE without background music (n = 20)	>18 (1. 27.15, 2. 25.40)	1 session - individual	- SUDS - APQ - STAI-Y - VR questionnaire - EDA - SCL - ECG	Post: 1 > 2
30	Shiban *et al*., 2017 ^44^	Others	Flying (community)	1: VRE with diaphragmatic breathing (DB) (n = 15) 2: VRE without DB (n = 14)	20–65 (1. 34.3, 2. 43)	1 session - 75 min - individual	- FFS - ASI	Post 1 = 2 1 week 1 = 2 1 year 1 = 2
31	Siegel *et al*., 2017 ^45^	Others	Spiders (community)	1: VBE to masked spiders (n = 30) 2: VBE to masked flowers (n = 30)	Not stated (19.6)	1 session - individual	- FSQ - BAT - SUDS	Post: 1 > 2
32	Steinman *et al*., 2014 ^46^	Others	Heights (community)	1: CBM (n = 27) 2: CBM + exposure (n = 27) 3: Exposure (n = 28) 4: Control (n = 28)	18–67 (23.58)	2 sessions - individual	- HIQ - BAT - AQ-anxiety	Post: 2 > 1 > 3 > 4 1 month: 1 = 2 > 3 = 4
33	Telch *et al*., 2017 ^47^	Others	Spider/snakes (community)	1: Fear reactivation prior to exposure therapy (n = 15) 2: Fear reactivation after exposure therapy (n = 17)	18–40 (21.31)	6 sessions - 3 min - individual	- BAT	Post: 1 = 2 1 month: 1 > 2

4DSQ, Four-Dimensional Symptom Questionnaire; APQ, Autonomic Perception Questionnaire; AQ, Acrophobia Questionnaire; AR, augmented reality; ASI, Anxiety Sensitivity Index; ATHQ, Attitude Towards Heights Questionnaire; BAT, Behavioural Approach/Avoidance Task; CBM, cognitive bias modification; CBT, cognitive behavioural therapy; CIDI, Composite International Diagnostic Interview; DFS, Dental Fear Scale; DPSS-12, Disgust Propensity and Sensitivity Scale-Revised; ECG, electrocardiogram measure; EDA, Electrodermal Activity; EmetQ, Emetophobia Questionnaire; EPDS, Edinburgh Postnatal Depression Scale; FAS, Flight Anxiety Situations Questionnaire; FAM, Flight Anxiety Modality Questionnaire; FFI, Fear of Flying Inventory; FFS, Fear of Flying Scale; FOBS, Fear of Birth Scale; FSQ, Fear of Spiders Questionnaire; HAI, Health Anxiety Inventory; HIQ, Heights Interpretation Questionnaire; HR, Heart Rate; iTBS, Intermittent Theta Burst Stimulation; MAMA, Maternal Adjustment and Maternal Attitudes; MDAS, Modified Dental Anxiety Scale; MFS, Medical Fears Survey; PHQ-9, Patient Health Questionnaire; SBQ, Spider Beliefs Questionnaire; SCL, skin conductance level; SDS, Sheehan Disability Scale; SFQ, Storm Fear Questionnaire; SPQ, Spider Phobia Questionnaire; STAI-Y, State Trait Anxiety Inventory – Form Y; SUDS, Subjective Units of Discomfort; TAU, treatment as usual; TES, Traumatic Event Scale; TSK, TAMPA Scale for Kinesiophobia; VAS-A, Visual Analogue Scale for Anxiety; VBE, very brief exposure; VRE, vancomycin-resistant Enterococci; VRET, virtual reality exposure therapy; WAQ, Weekly Anxiety Questionnaire; W-DEQ, Wijma Delivery Expectancy/Experience Questionnaire.

## Psychotherapy (n = 11)

Psychotherapies, specifically exposure-based therapies, have traditionally been considered the most robust treatment for most specific phobias ^3, 20^. Based on the inclusion criteria, 11 studies were included in this section on psychological therapies for specific phobia. The psychotherapies include cognitive behavioural therapy (CBT), systematic desensitisation, EMDR, exposure in different variations (very brief exposure, exposure online, hyper-real exposure and so on), psychoeducation, relaxation, applied muscle tension (AMT) and haptotherapy. Although CBT consists of a behavioural intervention component similar to exposure, it includes an additional cognitive component to change maladaptive beliefs. A plethora of specific phobias—including emetophopia (fear of vomiting), kinesiophobia (fear of movement), aerophobia (fear of flying), fear of childbirth, dental anxiety, spider phobia and fear of needles—were treated in these studies. Two out of the eleven studies had participants from a clinical sample ^21, 22^, whereas the rest of the studies obtained participants from the community ^10, 11, 13, 15– 17, 23– 25^. Given the exponential growth of internet usage in the last decade, computer/internet-based therapies are becoming more common ^26^. Unsurprisingly, two out of the five studies on CBT delivered interventions by using computer/internet-based programs ^16, 17^, whereas another two studies conducted the exposure of feared stimulus by using computer images ^24, 25^.

CBT was found to be superior to wait-list control and standard care control in all of the studies analysed. CBT was found to be significantly more efficacious than the control group with a large effect size ( *d* = 1.53) for emetophobia ^21^ and to provide better outcome than standard care for kinesiophobia in a 6-month follow-up ^11^. Internet-based CBT reduced the levels of childbirth fear significantly when compared with standard care of counselling by trained midwives ^16^. A single 1-hour session of computer-based CBT with psychoeducation and exposure components was found to significantly reduce dental anxiety in a 1-month follow-up as compared with wait-list control ^17^. A novel study that made a three-way comparison between CBT with systematic desensitisation ( *d* ranges from 1.32 to 2.23), CBT with EMDR ( *d* ranges from 1.23 to 2.67) and CBT with VR exposure therapy (VRET) ( *d* ranges from 1.11 to 2.55) for aerophobia found that all three hybrid interventions were efficient in reducing self-reported measures of the fear of flight ^22^.

Three studies examined different unique and novel modalities of exposure for the treatment of spider phobia. Cougle *et al*. ^10^ evaluated contamination-focused exposure as a prospective transdiagnostic treatment strategy for disgust-based fears and found marginally significant results only for participants with high levels of pre-treatment disgust propensity. The authors concluded that more studies were needed given that their study was possibly the first examination of a transdiagnostic contamination-focused exposure. As opposed to conducting the usual time-consuming exposure interventions, Siegel and Gallagher ^25^ tested out the effects of very brief exposure (VBE) of 25 spider images (of 33 milliseconds each) on spider phobia and found a significantly reduced avoidance of the tarantula as compared with the flower image control. Last but not least, hyper-real image exposure (real images exaggerated to increase fear) was found to have no added advantage as compared with real image exposure for spider phobia, and both were significantly superior to wait-list control ^24^. These studies represent innovative efforts to improvise on the well-researched exposure techniques for specific phobia in recent years.

Other than CBT and exposure therapies, different types of psychological treatments were found to be useful for particular specific phobias. A study for needle phobia found that AMT could significantly increase cerebral oxygenation and end-tidal carbon dioxide (both implicated in syncope, a risk specific to needle-related fear) as compared with slow breathing respiration intervention and no treatment control ^23^. For the fear of childbirth, group psychoeducation with relaxation exercises was found to improve maternal adjustment and childbirth experience as compared with the control group in a 3-month follow-up ^13^. Finally, haptotherapy, which aims to improve perception and attitude towards pregnancy and childbirth, was found to be an efficient new intervention for severe childbirth fear as compared with internet psychoeducation and care as usual ^15^.

In sum, evidence in the last five years continues to support CBT and exposure as effective interventions for a variety of specific phobias in both traditional and modern cyber modalities. Some researchers have attempted to improve existing interventions with improvisions, whereas other researchers explored novel treatment methods, both with varying levels of success. The journey to improving psychotherapies for specific phobia should continue in this right direction.

## Technology-assisted therapy (n = 7)

This section reviews technology-assisted therapies in the treatment of specific phobia. Technology-assisted therapies have received significant interest in the last decade because of their acceptability and increasing ease of application; they mitigate the typical aversion associated with *in vivo* exposure, and the efficacy is generalizable to real life ^48^. Seven of the studies fit the criteria: six used a VR platform and one examined the effectiveness of augmented reality (AR). In VRET, an individual with phobia experiences the fear situation in a fully artificial setting simulated by a computer program through a headset ^49^. Conversely, AR creates an immersive environment where the target feared stimulus is digitally enhanced and combined with other aspects in the real-life environment ^27^.

The seven VR and AR interventions reviewed were administered individually and addressed a variety of specific phobias, including phobias of heights, spiders, cockroaches, dental procedures, storms and movements. Participants in most of these studies did not have a clinically diagnosed phobia and instead were assessed to meet study inclusion criteria through a self-reported, pre-determined cut-off of subjective fear score. Five of the studies had active comparisons: two involved *in vivo* exposure ^27, 31^, one consisted of psychoeducation and progressive muscle relaxation ^30^, one of psychoeducation ^29^, and one of traditional physiotherapy ^33^. The remaining two studies compared VRET with a non-active control condition ^28, 32^. Treatment was typically a single session but in two studies this was not the case ^28, 33^. Outcomes were assessed through a range of self-report questionnaires and behavioural avoidance tests. Treatment outcomes at follow-up were reported by five studies, and the duration of the follow-up ranged from 1 to 52 weeks.

Two studies suggest that VR and AR exposure was associated with less improvement compared with *in vivo* exposure therapy, at least immediately after intervention. Miloff *et al*. ^31^ compared a single-session VRET with *in vivo* exposure in a non-inferiority trial involving 100 participants with a clinically diagnosed spider phobia. The authors found that although VRET significantly reduced behavioural avoidance ( *d* = 1.49) to a large effect size, it was not more effective than *in vivo* exposure in the treatment of spider phobia measured at the end of treatment. Non-inferiority was achieved only at 3 and 12 months after treatment. Drop-out rate was low for both conditions; 94% of the VRET group and 88% of the *in vivo* group completed the assessment at the 12-month time point. Similarly, a second study reported that reduction in spider or cockroach avoidance-related behaviour was more evident in the control *in vivo* exposure condition instead of the hypothesised AR exposure therapy treatment condition ^27^. At the 3- and 6-month follow-up, treatment efficacy was maintained and comparable across the two groups. Attrition rate was not significantly different, although participants in the AR condition had expected the intervention to be “less aversive”.

The remaining three studies with active control groups consistently established VRET as the superior treatment option. In one VRET-based intervention, participants who were exposed to a hierarchy of five dental anxiety scenarios (for example, a virtual dentist holding a syringe) had significantly reduced fears and avoidance behaviour compared to those who had only received standard information about dental anxiety through a pamphlet ^29^. The positive treatment effects were replicated at 1-, 12-, and 24-week follow-up. More importantly, 77% of participants in the VRET condition compared with 50% in the control condition ( *P* >0.05) underwent an actual dental procedure within 6 months of the intervention, indicating generalisability of the VRET treatment gains. Lima *et al*. ^30^ examined VRET in comparison with progressive muscle relaxation (PMR) and psychoeducation in the attenuation of storm fears. VRET was superior to PMR and psychoeducation in terms of the subjective units of distress experienced by the participants (measured after treatment). A separate outcome measure—that is, the Storm Fear Questionnaire (SFQ)—was used to assess treatment effects 1 month after the intervention; the authors reported that treatment gains were maintained and that the two groups performed comparatively. When VRET was used as an adjunctive intervention, it improved the effects of traditional physiotherapy among participants with kinesiophobia (fear of movements) ^33^.

Furthermore, VRET delivered a consistently better outcome than a non-treatment control condition in two studies. In the first, participants who underwent six 30-minute sessions of VRET had a greater reduction of distress, anxiety, and avoidance scores on the Height Interpretation Questionnaire (HIQ) in comparison with those who were assigned to treatment as usual (in this case, the team quantified as equivalent to “receiving no treatment”) ^28^. This was maintained at 4-week follow-up. Another group of researchers demonstrated that VRET treatment was more efficacious in decreasing subjective fear of spiders and related avoidance behaviour compared with a wait-list condition ^32^. As in other studies, the between-group effect size was large ( *d* = 0.85). In addition, there was no attrition reported in the VRET group, suggesting good tolerability of the treatment.

Overall, the evidence reviewed suggests that VRET is a robust and well-tolerated intervention for a range of specific phobias among adults. The positive treatment effects of VRET were maintained mostly at follow-up periods. It should be noted that the immediate efficacy of VRET in relation to *in vivo* exposure, which is the gold standard in the treatment of specific phobia, may not be superior but the two studies ^27, 31^ went on to determine that the longer-term gains were equivalent.

## Pharmacotherapy (n = 6)

The general view is that medications are of limited benefit in the treatment of specific phobia ^50, 51^. One review of treatment for phobias in adults, published in 2007, concluded that medication has limited use but that D-cycloserine could be promising as an adjunctive treatment ^3^. Not surprisingly, our search yielded only six studies investigating pharmacological treatment among adults with specific phobia. In addition, these articles had investigated the potential role of various compounds as adjuncts to psychotherapy.

Meyerbröker *et al*. studied the use of yohimbine and propranolol to enhance the effect of VRET in participants with specific phobia ^35^. Yohimbine hydrochloride was postulated to enhance emotional memory by elevating norepinephrine level, whereas propranolol could reduce psychophysiological arousal and make exposure therapy more tolerable. Study participants were randomly assigned to receive yohimbine, propranolol or placebo an hour before exposure therapy. The study did not find differences among the three treatment groups. In addition, the propranolol group reported a higher anxiety level 3 months later in comparison with post-intervention. In a similar double-blind, block-randomized, placebo-controlled ******** study, 20 mg of hydrocortisone or placebo was administered an hour before exposure therapy for participants with spider phobia over two therapy sessions ^12^. Glucocorticoids was hypothesised to promote the fear extinction process ^52^. Those who received cortisol before exposure-based group therapy did not show immediate post-treatment improvement but reported significantly greater reduction in spider phobic symptoms 1 month later. The authors hypothesised that time was needed for the glucocorticoid effects on the consolidation processes to take place.

Several trials investigated the effect of post-exposure administration of agents that could enhance fear extinction. Methylene blue could improve memory consolidation and enhance the effect of exposure therapy ^53^. Telch *et al*. ^38^ randomly assigned 42 participants with claustrophobia to receive methylene blue or placebo following extinction training. In total, 260 mg of methylene blue was administered in three equally divided doses immediately after training, 6 to 10 hours later and again 6 to 10 hours later. The group receiving methylene blue showed greater improvement at post-treatment assessment but fared worse 1 month later. A separate clinical trial investigated the use of propranolol to disrupt the process of memory reconsolidation during exposure therapy ^37, 54, 55^. Forty-five participants (18 to 32 years old) with spider phobia were randomly assigned to receive propranolol or placebo following exposure therapy or just propranolol alone. For the intervention group, 40 mg of propranolol was administered after a short exposure to a tarantula. After treatment, the intervention group performed better on behavioural assessments than the other two groups, even at the 3-month and 1-year follow-up. However, the self-reported outcomes showed significant improvement in the intervention group only at the 3-month and 1-year follow-up. The authors hypothesise that this suggests that cognitive change follows extinction of the fear behaviour. The effect of post-exposure cortisol administration was investigated in a double-blind placebo-controlled trial involving 43 subjects with spider phobia ^36^. Cortisol did not add further benefit to exposure therapy and was found to have an adverse effect on fear renewal on the basis of behavioural approach tests at the 7-month follow-up.

A final agent investigated for possible enhancement of benefit of exposure therapy is intranasal oxytocin ^34^. Oxytocin is a mammalian neuropeptide that modulates activity of the neuro-circuit mediating fear extinction and memory processes and may enhance the fear extinction effect of exposure therapy if administered prior ^56, 57^. This small study involved 23 patients, and those who received oxytocin prior to exposure therapy fared worse than those who received placebo, suggesting that intranasal oxytocin was not beneficial.

Among the pharmacological agents investigated, there were positive preliminary data for propranolol and glucocorticoid as adjunctive treatment to psychotherapy. Of particular interest is propranolol as the timing of its administration in relation to the exposure therapy appeared to be important to its efficacy. Overall, further studies are needed to see whether these positive results can be replicated.

## Others (n = 9)

The remaining nine studies, which investigated an intervention that did not fit clearly into the scope of a psychological, technological or pharmacological treatment, are reviewed here. A specific cluster of the treatment modality targeted a different aspect of what is understood in the development and the subsequent extinction of phobias: the pathological fear seen in phobias is conceptualised to arise from an imbalance between the amygdala activation to non-threatening stimuli and the prefrontal cortex (PFC) (inhibitory effect on the amygdala) ^58^.

Notzon *et al*. ^42^ examined the use of intermittent theta burst stimulation (iTBS) to regulate the PFC and improve phobic symptoms. iTBS is a more intense form of transcranial magnetic stimulation (TMS). A single session of iTBS administered prior to immersion in a VR spider scene showed no effect on the subjective reactions provoked by VR. It was uncertain whether more sessions would be more effective or whether this TMS protocol was appropriate. Surprisingly, with iTBS, participants demonstrated an increase in sympathetic response on electrophysiological parameters and this was possibly due to increased attention towards the presented stimuli ^59^.

Psychophysiological studies have shown that autonomic responses (for example, fear responses) can be conditioned without awareness ^60, 61^. Hence, researchers have investigated whether autonomic fear arousal can be bypassed through the masking of subjective awareness to a feared object and consequentially emotional distress that typically accompanies exposure therapy is limited. Siegel *et al*. ^45^ evaluated the use of VBE therapy in treating spider phobia. Skin conductance levels (SCLs) were used as a measure of sympathetic activity. After a single session, VBE reduced avoidance behaviour without increasing SCL or subjective distress. The lower the SCL during VBE, the more it reduced fear. The authors hypothesised that effective masking and reduction in distress weakened the association between the phobic stimulus and fear response, which thereby facilitated an automatic process of fear reduction.

Phobias can alter and distort the realistic appraisal of the feared stimulus which consequently creates a vicious cycle that perpetuates fear ^62^. Thus, Dreyer-Oren *et al*. ^39^ and Steinman *et al*. ^46^ evaluated the role of cognitive bias modification (CBM) in changing perceptual bias in height phobia. Both studies had a CBM intervention group, a CBM with exposure therapy group, an exposure therapy-only group, and a control group. Despite only a couple of intervention sessions, all four groups showed improvement in perceptual bias, interpretation bias, and height fear symptoms at 1-month follow-up. However, those who underwent CBM or exposure therapy experienced this improvement earlier (that is, immediately after intervention). The fact that CBM and exposure therapy had similar effects on interpretation bias reflects how different pathways to fear reduction can result in similar cognitive change.

A few other studies have looked into how fear memories can be altered. When a specific memory trace is retrieved from long-term memory, it exists in a labile state that is more amenable to correction before it reconsolidates again ^63, 64^. Telch *et al*. ^47^ studied the effect of giving a reactivation cue 30 minutes prior to *in vivo* exposure therapy for spider or snake phobia. After one session, they did not find any difference in avoidance scores between intervention and control groups after treatment. At the 1-month follow-up, the intervention group reported lower peak fears but still no difference in expected fear in comparison with controls.

Maples-Keller *et al*. ^40^ studied the effect of giving a reactivation cue 10 minutes prior to VRET for flying phobia. Participants underwent four sessions of VRET, and cues were shown prior to each session. Those who received a reactivation cue had similar reduction in fear symptoms at post-treatment and at 1-year follow-up in comparison with those in the control group. However, the control group demonstrated increased physiological arousal to the feared exposure compared with the reactivation group. Although memory reactivation prior to exposure therapy may have a limited impact on reported fear symptoms, they may be an effective adjunctive to exposure therapy based on physiological outcomes.

In phobia treatment, a form of relaxation is usually combined with exposure therapy to aid in systematic desensitisation of fear. Two studies looked at using music and diaphragmatic breathing to augment exposure therapy. In Seinfeld *et al*. ^43^, participants were immersed in VR scenarios that induced fear of heights while music played in the background. Anxiolytic effects were significant only after the experience but not during the experience. Therefore, music may be useful only in certain phases of therapy. Shiban *et al*. ^44^ used diaphragmatic breathing to augment VRE for flying phobia. At both 1 week and 1 year after intervention, a significant reduction of fear was confirmed by a self-report measure in both intervention and control groups, and there was no significant difference between groups. Limitations included the lack of differentiation between the types of aviophobia and uncertainty over the appropriateness of the VRE therapy protocol.

The review also yielded a study that investigated relaxation in the treatment of blood-injection-injury phobias. Meuret *et al*. ^41^ compared the efficacies of Symptom Associated Tension (SAT) Training and Hypoventilation Respiratory Training (HRT) with that of Relaxation Skills Training (RST) in treating blood-injection-injury phobias. These were delivered via 12-minute video-guided trainings. Both SAT and HRT showed a reduction in phobic symptoms in comparison with RST through self-report. In addition, HRT reduced ventilation, increased pCO _2_ (partial pressure of carbon dioxide) and elevated blood pressure throughout exposure and recovery phases. This might be useful in preventing the fainting spells sometimes associated with blood-injection-injury phobias.

In conclusion, CBM, VBE therapy, and memory retrieval extinction have shown some efficacy in the treatment of specific phobias. iTBS and the use of music or diaphragmatic breathing to augment exposure therapy have shown limited benefits.

## Discussion

This rapid review sought to provide a systematic update of the current development in the treatment of specific phobias within the adult population in the last five years. It also attempted to summarise the efficacy evidence associated with these interventions. In total, 33 studies met the inclusion criteria and were included in the review.

## Summary of main findings

First, results from a number of the psychotherapy-related studies reported CBT, implemented either independently or as an adjunctive, to be a superior intervention with large effect sizes. This is unsurprising as cognitive factors (for instance, the irrational overappraisal of danger to a specific fear stimulus ^65^) have been conceptualised and implicated in the development and maintenance of specific phobias ^66– 68^. Exposure therapy is an empirically supported and widely accepted treatment of choice for phobias ^69^. However, none of the psychotherapy-related studies had its efficacy evaluated against exposure therapy as an active comparison group. A previously published meta-analysis did conclude that CBT did not outperform *in vivo* exposure therapy ^20^. The length of psychological interventions varies from one to 12 sessions, depending on the treatment modality. In general, CBT which consists of both cognitive restructuring about the phobic beliefs and exposure tends to require more sessions (10 to 12 sessions; for example, ^21, 22^). In contrast, intervention programs involving pure behavioural techniques such as AMT ^23^, very brief exposure ^25^ and most technology-assisted therapies ^29, 30, 32^ are typically brief (one or two sessions) and have been associated with improvement in phobia symptoms compared with the respective control conditions.

Sufficient evidence reviewed earlier suggest that technology-assisted therapies, designed to decrease fear and avoidance behaviour in adults with phobia by combining VR or AR platforms (or both) with an exposure paradigm, were demonstrated to be generally efficacious. This is consistent with existing evidence presented in previous meta-analyses for VRET ^48, 70^. In addition, attrition rates were low ^31^, and the majority of the study participants reported VRET to be a less aversive treatment alternative ^32^, further corroborating the overall acceptability. Third, pharmacological agents, including yohimbine, propranolol, hydrocortisone and oxytocin, have been investigated in the last five years exclusively as an adjunctive treatment to *in vivo* exposure. Of particular interest are propranolol and hydrocortisone, which have mixed findings. The timing of administration appears to be important: glucocorticoid may be beneficial if given before exposure whereas the converse is true for propranolol. Propranolol, when administered after memory activation or when time was allowed to consolidate its effect, may enhance fear extinction and reduce physiological arousal.

A number of alternative and novel treatment options that sought to modify the cognitive pathway of activated fear responses and memories have gained empirical attention in the last five years. Initial findings from studies involving CBM and VBE protocols appear to provide some promising outcomes in the context of a significant mitigation of self-reported fear, associated physiological responses, and cognitive biases to the specific phobia.

Last but not least, the majority of the studies recruited participants from the community who were assessed or self-reported (or both) to have a significant degree of impairment from a phobia; only three of the studies included individuals from clinical populations ^21, 22, 29^. Persons with specific phobias are mostly hesitant to seek treatment because of the prospect of having to confront their fears during the intervention process and do not commonly present for intervention at clinical settings ^71^. Hence, although most of the pooled participants were community-based and this may, to an extent, limit the generalizability of the findings to a clinical population, the sample characteristics appears reflective of the trend in the treatment-seeking behaviour of those with specific phobias.

## Challenges and implications

One of the major issues faced in this rapid review relates to the heterogeneity of specific phobias. A plethora of phobias were addressed, ranging from the more prevalent ones such as heights and spiders to the more uncommon ones such as storms and vomiting. Also, a wide variety of self-report questionnaires, behavioural avoidance tasks, and physiological measures were administered to determine outcomes. This general lack of uniformity complicates the process of organising the pooled findings into a coherent and meaningful overview that encapsulates the true efficacy of specific interventions for clinicians and researchers interested in specific phobias. The heterogeneity further precludes the use of a meta-analysis to quantify findings.

A limited number of interventions were identified for each category of phobia. Extra attention was given to describe and differentiate the treatment responses. Despite this, the number of good-quality and robust interventions catering to each specific type of phobia needs to be substantially increased before a conclusive outcome can be made on whether a particular treatment was useful and effective in reducing phobia-related difficulties. A number of the studies did not have sufficient sample sizes and therefore were not powered to adequately conclude whether the improvements were directly associated with the intervention or due to chance or extraneous factors not measured in the studies. A replication of these studies with larger sample sizes and with treatment-seeking clinical populations would strengthen the current evidence demonstrated in the studies reviewed.

In addition, the included studies often made use of validated self-report questionnaires to characterise the participants’ levels of fear and eligibility in the majority of the studies; that is, participants had to score above a non-standardised cut-off to qualify for enrolment. Although the present review excluded any studies with participants who were not representative of the targeted phobia population (that is, analogue studies), which improved the reliability of the findings presented, such evaluation approaches may not have the same rigour as standardised structured clinical diagnostic assessments. It is equally critical to point out that many of the studies recruited participants through community avenues. This could imply that the treatment options reviewed may not have the same effectiveness for individuals who experience impairment presenting in clinical settings.

In consideration of the aims to provide a timely update of the latest evidence associated with treatment of specific phobia to inform research and clinical practice and to reduce the resource-intensive nature of systematic reviews, this study adopts a rapid review methodology. A rapid review can be regarded as an alternative type of systematic review but with some of its associated principal components adapted or simplified (or both) ^72^. Therefore, to an extent, rapid reviews can be susceptible to biases. For instance, only two databases were included in the search, and dissertation, grey literature, and studies without a control condition were also excluded. Hence, the results presented may not fully account for the range and effectiveness of available phobia-related interventions. It should be pointed out that the inclusion and exclusion criteria decided *a priori* maintained a level of uniformity across the included studies for comparison. Also, the present review did not assess the quality of the studies by using an established checklist. Indicators of bias such as the role of sponsors in studies and inconsistencies or any missing information in reporting were not systematically verified for. Another possible bias relates to the issue that studies with non-significant results are less likely to be published (that is, publication bias), which can create an overestimation of the actual efficacy of a type of treatment for specific phobias.

Last but not least, although follow-up outcomes were reported by most of the studies and overall were quite promising, only a couple of studies assessed whether the treatment was transferable to real life ^29^. It is unknown whether the effectiveness of the treatment, administered under controlled research settings, led to actual improvement in the participants’ functioning and quality of life (for instance, being able to go through a dental procedure or successfully taking a flight).

## Conclusions

Findings collated from the studies published in the last five years included in this systematic review are largely congruent with the existing evidence base of specific phobia; psychotherapy (in particular, CBT) remains one of the interventions associated with more substantial positive treatment outcomes. Technology-assisted treatment options such as VRET have also been found to be efficacious and seemingly non-inferior to traditional exposure therapy. It is necessary to encourage future trials involving technology-assisted treatments to include a feasibility analysis of infrastructure and cost to facilitate routine implementation in mental health-care settings. Alternative treatments involving cognitive modification or masking of fear associated with specific phobias are likely to receive continued empirical attention and can help individuals to become less resistant to the treatment. However, the practical applicability within a routine clinical or community setting (or both) requires further investigation. Challenges associated with the inherent heterogeneity of specific phobias need to be overcome in order for a more focused conclusion on treatment and its efficacy.
